# Prevention of peripherally inserted central line-associated blood stream infections in very low-birth-weight infants by using a central line bundle guideline with a standard checklist: a case control study: Prevention of PICC catheter related infections

**DOI:** 10.1186/s12887-015-0383-y

**Published:** 2015-06-18

**Authors:** Wei Wang, Chunling Zhao, Qinglian Ji, Ying Liu, Guirong Shen, Lili Wei

**Affiliations:** Departments of Neonatal, The Affiliated Hospital of Qingdao University, Qingdao, 266003 China; Departments of Central Supply Service, The Affiliated Hospital of Qingdao University, Qingdao, 266003 China; Departments of Radiology, The Affiliated Hospital of Qingdao University, Qingdao, 266003 China; Departments of Nursing, Weifang Nursing Vocational College, Qing zhou, 262500 China; Departments of Humanities Nursing, Qingdao Medical College, Qingdao, 266021 China; Departments of Nursing, The Affiliated Hospital of Qingdao University, Qingdao, 266003 China

**Keywords:** Central line bundle, Checklists, Very low birth weight infant, PICC, Catheter related infection

## Abstract

**Backgrounds:**

Catheter-related infections (CRIs) are one of the severe complications of PICC placement. If treatment is not timely or correct, the incidence of infection and mortality rate can be high. A central line bundle (CLB) guideline was first proposed by the Institute for Healthcare Improvement, and included five key measures. Very low-birth-weight infants (VLBWIs) have a low immune response and indistinct symptoms after infection compared with other populations (Costa P, Kimura AF, de Vizzotto MP, de Castro TE, West A, Dorea E. Prevalence and reasons for non-elective removal of peripherally inserted central catheter in neonates. Rev Gaucha Enferm. 2012;33:126–33). Some reviews have focused on the effect and safety of a CLB in VLBWIs and its preventive effect on bacterial colonization and infection.

**Methods:**

Fifty-seven VLBWIs who underwent PICC insertion at a hospital in Qingdao, China, between November 2012 and June 2013, and for whom a CLB guideline and a standard checklist were adopted, were included in the CLB group. In contrast, 53 VLBWIs who underwent PICC insertion, but for whom a CLB guideline and a standard checklist were not adopted, were included in the control group. The incidence of CRIs was compared between before and after the treatment.

**Results:**

The incidence of infection showed a statistically significant reduction from 10.0 to 2.20 per 1000 catheter days in the control group (*P* < 0.05). The incidence of catheter-related bloodstream infections decreased from 3.1 to 0 per 1000 catheter days, and that of colonization infections decreased from 6.9 to 2.2 per 1000 catheter days (*P* < 0.05), both of which indicated a statistically significant difference. The indwelling catheter time was 24.8 ± 7.4 days in the control group and 31.9 ± 15.0 days in the study group (*P* < 0.05), and these values were significantly different.

**Conclusion:**

The use of a CLB guideline with a standard checklist could be effective and feasible for preventing CRIs in VLBWIs and prolonging indwelling catheter time.

## Background

The use of the peripherally inserted central catheter (PICC) technology is widespread because of its simple operation, osmotolerance, and long indwelling time [1]. However, catheter-related infections (CRIs) are one of the severe complications of PICC placement, with incidence rates ranging from 16.4 to 28.8 % [2–4]. If treatment is not timely or correct, the incidence of infection and mortality rate can be high [5]. Therefore, knowledge on the manner in which such CRIs can be reduced or eliminated is important. A central line bundle (CLB) guideline was first proposed by the Institute for Healthcare Improvement, and included five key measures—namely, hand hygiene, maximum sterility, chlorhexidine skin disinfection, choosing the best puncture site, and daily assessment of whether to remove the catheter [6]. These measures have been proven to effectively reduce the occurrence of catheter-related bloodstream infections (CRBSIs) [7, 8].

Very low-birth-weight infants (VLBWIs) have a low immune response and indistinct symptoms after infection compared with other populations [9]. Some reviews have focused on the effect and safety of a CLB in VLBWIs and its preventive effect on bacterial colonization and infection [10, 11]. In the present study, we combined the CLB guidelines with actual CRBSI conditions to develop a plan for PICC insertion and maintenance and also used a standard checklist to ensure that each measure of the CLB guideline was implemented. The investigation of the effectiveness of the CLB guideline in preventing PICC CRIs in VLBWIs was always common, Pronovost et al [12] has conducted a collaborative cohort study in Michigan, and the results showed a sharply decreased of the catheter-related bloodstream per 1000 catheter-days decreased from 2.7 infections at baseline to 0 at 3 months after implementation of the study, so we would conducted a retrospective study to investigate the guidline use in China, to find the effectiveness and feasibility of central line bundle (CLB) guideline with a standard checklist in the prevention of peripherally inserted central catheter (PICC)-related infections (CRIs) in very low-birth-weight infants (VLBWIs).

## Methods

### Clinical data

In this case–control study, 110 VLBWIs who received PICCs were enrolled. All the patients met the standards of PICC insertion. The CLB group included 57 VLBWIs for whom the CLB guideline and a standard checklist were adopted between November 2012 and June 2013. In contrast, the control group included 53 patients in whom catheters were inserted, but for whom the CLB guideline and a standard checklist were not adopted. The two groups had no differences in gestational age, sex ratio, or birth weight (*P* >0.05, Table 1). This study was conducted in accordance with the declaration of Helsinki. This study was conducted with approval from the Ethics Committee of Qingdao University. Written informed consent was obtained from all parents.

**Table 1:** General comparison of patients with PICC

Group	Gestational age (w)	Sexuality		Birth-weight (g)
		Male	Female	
Control (n = 53)	30.8 ± 1.6	23	28	1186.1 ± 180.4
CLB (n = 57)	30.4 ± 1.9	30	29	1183.9 ± 207.7
t/*X* ^2^	1.093	0.363		0.058
*P*	0.277	0.547		0.954

### Bundle insertion

PICCs were inserted in all the participants by a senior nurse who was qualified to perform catheter insertion. The following conditions were established for the CLB group: 1) creation of a PICC treatment center, which based on a treatment trolley, always with sufficient instrument and drugs in it and be checked regularly, which was placed in the catheter lab, meantime qualified doctors and nurses were also needed. 2) hand hygiene, 3) maximum sterility, 4) skin preparation, and 5) selection of the best puncture site. The PICC supplies (single-lumen 1.9-Fr catheter and No. 26 catheter sheath, BD Inc., Illinois, USA) were kept in a fixed location, and regular inspections were undertaken to ensure the presence of adequate supplies and backups within the study period. The VLBWIs underwent PICC line insertion only in the treatment center. The nurse and assistants were required to wash their hands in strict accordance with the seven-step hand-washing method [13]. For maximum sterility, the nurse and assistants wore sterile surgical gowns, gloves, hats, and masks. Masks required complete and tight wrapping around the nose and mouth. The headcaps completely and tightly covered all the hair. The patients were completely covered with sterile towels from head to toe, with only the puncture site exposed. Skin preparation was performed by washing the prepuncture upper arm (from the fingertips to the fossa cubitalis) with warm soapy water before PICC insertion. A 75 % alcohol solution was used to clean and degrease the skin first. The procedure was repeated two or three times according to the patient’s skin condition. Thereafter, Anerdian was used to disinfect the armpits, down to the fingertips, and the procedure was repeated three times. Anerdian is a kind of skin disinfectants that used widely in clinics, in which contains iodine (0.2 ± 0.02 %), chlorhexidine acetate (0.45 ± 0.045 %), ethanol (65 ± 5 %). Anerdian could kill intestinal bacteria, pyogenic bacteria, yeast and pathogenic bacteria and many other bacteria that caused hospital infection. Manufacturer was disinfection technologies Ltd. on Haili Kang, ShangHai. The punctured area was dried naturally after disinfectant application. The best puncture site was then selected. The first choice was the basilica vein, followed by the cubital and axillary veins. Puncture of the lower limbs was avoided. In the control group, strict hand hygiene, skin preparation, and aseptic manipulation practices were followed; however, the measures of using the PICC treatment center, ensuring maximum sterility, or selecting the best puncture site were not adopted.

### Bundle maintenance

The following conditions were established for the CLB group: 1) hand hygiene, 2) dressing management, 3) filling and sealing the catheter tube, and 4) daily assessments by duty nurses. Hands were washed in strict accordance with the seven-step hand-washing method, or a hand disinfectant was used before and after touching the catheters and dressings. For dressing management, on the day after catheter insertion, then was replaced every week thereafter. Duty nurses undertook daily assessments as follows: observe whether the puncture site exhibited redness, swelling, tenderness, or inflammation; avoid removing the catheter because of simplex fever; and comprehensively evaluate the need to remove the catheter according to clinical manifestations and laboratory findings. When the catheter is no longer necessary, it should be removed in a timely manner.

The following conditions were developed for the control group: the group received routine nursing care, including the use of an aseptic technique, timely sealing of the catheter, and film replacement.

### Use of a standard checklist

In the study group, PICC insertion and maintenance were monitored by a nurse who was qualified to perform catheter insertion. Each measure was assessed according to the standard checklist of CDC. Any violation of the operating rules was stopped in a timely manner and corrected, and the accurate implementation of the measures was ensured. In the control group, the standard checklist was not used.

CRI diagnosis and classification criteria [14] were used to determine the following conditions: 1) local infection, defined as skin with redness, tenderness, or secretion around the intubation; 2) phlebitis, defined as painful and diffuse erythema occurring at the subcutaneous site along the catheter, which is not related to physical or chemical factors; 3) catheter colonization, defined when the insertion site had no signs of infection and the distal part of the catheter had pathogens amounting to ≥15 colony-forming units (CFU)/tablet, with semiquantitative cultures or pathogens amounting to ≥1000 CFU on the quantitative culture; and 4) CRBSI, defined when the same pathogen was isolated on quantitative or semiquantitative catheter cultures and other blood cultures, samples of which were obtained through venous drawing. The presence of the pathogen was accompanied by the clinical manifestations of blood infection, and there may have been other definite sources of blood infections in addition to the catheter.

### Sample collection

The patients without clinical CRI symptoms [15] underwent routine alcohol disinfection around the catheter three times when the catheter was removed, along with disinfection with Anerdian, which was also performed three times. The operator wore gloves and used sterile towels to form a sterile area, moved the catheter tip 5 cm into the sterile blood dish, and directly took the samples to the microbiological laboratory for semiquantitative catheter culture. Patient samples that were suspected of being infected were divided into reserved and nonreserved catheters.

The reserved catheter met the following conditions: the catheter blood and peripheral blood that were simultaneously extracted were further subjected to pathogen and antimicrobial susceptibility tests, and the time of blood sampling was ≤5 min. If the culture results confirmed or could not rule out infection, the catheter was removed immediately, and a catheter tip culture and a sensitivity test were performed. The nonreserved catheter met the following conditions: two peripheral blood samples were extracted for pathogen and catheter tip cultures, and an antimicrobial susceptibility test was performed.

### Outcome measures

The outcome measures for both groups were as follows: 1) Nurses observed and recorded the patients’ temperatures, partial oozing of blood, exudation, redness, swelling and indurations, and punctured limb swellings. Nonspecific clinical manifestations associated with infections, such as listlessness, jaundice, and persistent and recurrent apnea, were also noted. 2) Laboratory test results included catheter tip and blood cultures. The following formula was used to determine the presence of PICC-related infection [16]: cases of infection/PICC catheter days over the same period × 1000 %, expressed as 1000 catheter days. Since there may be many reasons causing removal of the PICC catheter, like mechanical problems with the hardware, physician/provider preferences, infection, etc., the Kaplan-Meier survival calculation was performed to find the survival differences between the control group and CLB group that caused by infection.

### Statistical analysis

Statistical analysis was performed using the SPSS software. The measured data were presented as mean ± standard deviation values, and the Student *t* test was used to compare data between the groups. Qualitative data were analyzed by using the chi-square test. A *P* <0.05 denoted a significant statistical difference.

## Results

The gestational age was 30.8 ± 1.6 weeks in control group, and 30.4 ± 1.9 weeks in CLB group, while the birth-weight was 1186.1 ± 180.4 g in control group and 1183.9 ± 207.7 g in CLB group, there was no significant differences were observed in gestational age, sex, and birth weight between the two groups (*P* >0.05, Table 1).

The cases of CRI in control group was 13 while in CLB group was 4, so the CRI infection rate was lower in the CLB group, and the indwelling catheter days was 24.8 ± 7.4 days in control group and 31.9 ± 15.0 days in CLB group, these differences were statistically significant (Table 2). PICC was an important venous access for VLBW, so the longer the time the better the outcome of the infants during the plan time. In the study, all the cases that removed the catheter were forced. In control group, there were 8 cases remove the catheter before the fit time, 4 cases because of infection, 3 cases because of catheter blockage and the other one was because of accident of the nurse; in CLB group, there were 3 cases, who were all because of catheter blockage.

**Table 2:** Comparison of CRI and the indwelling catheter days ($\overline{x}\pm s$)

Group	Total catheter days (d)	Cases of CRI cases	CRI infection rate	Indwelling Catheter days
			(/1000 indwelling catheter days)	
Control (n = 53)	1299	13	10.0	24.8 ± 7.4
CLB (n = 57)	1819	4	2.2	31.9 ± 15.0
*x* ^2^/t′			8.522	3.326^a^
*P*			0.004	0.001

^a^Population variance heterogeneity of indwelling time adopted correction of *t* test

The colonization infection case was 9 in control group and 4 in CLB group, CRBSI was 4 in control group and 0 in CLB group, and these differences were statistically significant (*P* <0.05; Table 3).

**Table 3:** Comparisons of CRBSI and colonization infection rate (Cases, / 1000 catheter days)

Group	Total catheter days (d)	Colonization infection		CRBSI	
		Cases	Infection rate	Cases	Infection rate
Control	1299	9	6.9	4	3.1
CLB	1819	4	2.2	0	0
*x* ^2^			4.082		3.463^a^
*P*			0.043		0.063

^a^There was a cell-expectation count as less than 5, the minimum expectation count was 1.67, using Variance test to correct

**Fig. 1:**
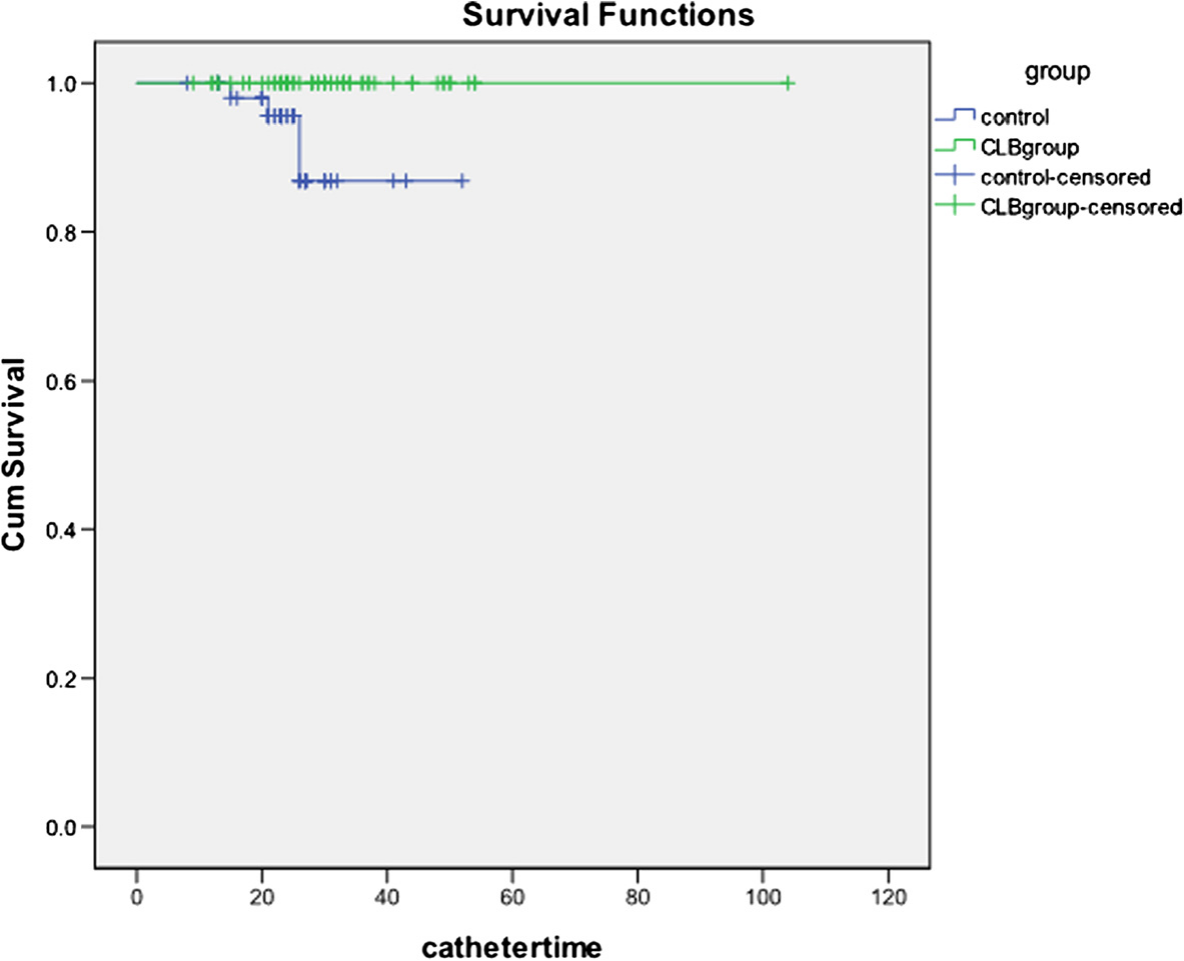
Survival rate in 2 groups

The survival rate in CLB group was higher than that in control group, which showed in Fig. 1 (*χ*^2^ = 5.484, *P* = 0.019).

## Discussion

This study shows that the CLB guideline with the standard checklist effectively reduced the incidence of colonization infections and CRBSIs in the VLBWIs. The incidence of CRIs decreased from 10.0 per 1000 catheter days before the intervention to 2.2 per 1000 catheter days after the intervention (*P* <0.05), and the indwelling catheter time increased from 24.8 ± 7.4 to 31.9 ± 15.0 catheter days (*P* <0.05). Furthermore, colonization infections decreased from 6.9 to 2.2 per 1000 catheter days (*P* <0.05). The number of laboratory studies on catheter colonization infections was small, mainly because no symptoms could be observed after the onset of infection. Moreover, we did not perform a catheter tip bacteria culture when the catheter was removed; thus, the detection rate was low [17]. In the present study, all the removed catheters were subjected to catheter tip culture, where we found the highest colonization infection rate. If we had not been practicing advanced prevention in our institution, this might have been a potential risk factor of CRBSI [18].

The CRBSI rate in the control group was 3.1 per 1000 catheter days. The incidence of CRBSI among the newborn infants was 1.6–18.5 per 1000 catheter days [19–21], which is not significantly different compared to that of the control group; however, the incidence in the control group subsequently decreased to 0. Four cases in control group had catheter-related bloodstream infections which lead to extubation, while there was none in the CLB group. This rate might be related to the implementation of the CLB with the standard checklist.

This study not only adopted the five measures put forward by the Institute for Healthcare Improvement but also added measures—after searching the literature, consulting with experts, and connecting this information with good practice—namely, establishing a PICC treatment center, dressing management, and correctly filling and sealing tubes. Each measure was evidence based. Furthermore, other studies had indicated that operator compliance monitoring should be part of the strategy to prevent nosocomial infection and could prevent infection to a large extent [22, 23].

Consequently, this study employed quality control nurses to monitor the entire process of PICC insertion and assess whether the measures were being implemented according to the checklist, which also included timely remedies to ensure compliance with the rules of the operation. The criteria were placed on cards, which were placed next to the incubators where the PICC patients were confined. These reminded nurses to maintain catheters according to the guidelines, by using the standard operations, and to avoid using subjective experience, omitting implementations, and errors.

### Limitation

The sample size was not enough to gain a powerful conclusion since PICC was started late in the hospital, also the immunity differences of the included infants may lead to bias.

## Conclusions

In summary, a CLB guideline can effectively, simply, and feasibly reduce the incidence of colonization infection and CRBSIs in VLBWIs, without any additional investment. In addition, this study found that it is important to ensure that each measure is completed. We found that each measure was implemented effectively by following the standard checklist, and hence, the CLB guideline could have a greater impact in preventing infection.
